# The cholinergic system modulates negative BOLD responses in the prefrontal cortex once electrical perforant pathway stimulation triggers neuronal afterdischarges in the hippocampus

**DOI:** 10.1177/0271678X211049820

**Published:** 2021-09-30

**Authors:** Alberto Arboit, Karla Krautwald, Frank Angenstein

**Affiliations:** 1 Functional Imaging Group, Deutsches Zentrum für Neurodegenerative Erkrankungen (DZNE), Magdeburg, Germany; 2 Leibniz Institute for Neurobiology (LIN), Magdeburg, Germany; 3 Medical Faculty, Otto von Guericke University, Magdeburg, Germany

**Keywords:** Cerebral blood volume (CBV), electrophysiology, negative BOLD response, pilocarpine, scopolamine

## Abstract

Repeated high-frequency pulse-burst stimulations of the rat perforant pathway elicited positive BOLD responses in the right hippocampus, septum and prefrontal cortex. However, when the first stimulation period also triggered neuronal afterdischarges in the hippocampus, then a delayed negative BOLD response in the prefrontal cortex was generated. While neuronal activity and cerebral blood volume (CBV) increased in the hippocampus during the period of hippocampal neuronal afterdischarges (h-nAD), CBV decreased in the prefrontal cortex, although neuronal activity did not decrease. Only after termination of h-nAD did CBV in the prefrontal cortex increase again. Thus, h-nAD triggered neuronal activity in the prefrontal cortex that counteracted the usual neuronal activity-related functional hyperemia. This process was significantly enhanced by pilocarpine, a mACh receptor agonist, and completely blocked when pilocarpine was co-administered with scopolamine, a mACh receptor antagonist. Scopolamine did not prevent the formation of the negative BOLD response, thus mACh receptors modulate the strength of the negative BOLD response.

## Introduction

Acetylcholine (ACh), via nicotinic ACh receptors, is a fast acting neurotransmitter, but via muscarinic ACh receptors, it is a modulatory neurotransmitter that controls neuronal excitability, presynaptic neurotransmitter release and coordinates the firing of groups of neurons.^1^ The central cholinergic system is involved in many cognitive functions, such as attention to sensory stimuli, coding of location and movement, learning, and memory,^2–4^ as well as in various neurological disorders including depression, Alzheimer’s disease, schizophrenia, and epilepsy.^5–8^

The hippocampus and the prefrontal cortex are known to be modulated by ACh; the majority of the cholinergic projections targeting these areas originate in the basal forebrain complex (medial septum).^9,10^ Besides receiving cholinergic projections from the medial septum, the hippocampus and the prefrontal cortex are also functionally connected, i.e., the hippocampus projects heavily to the (ventral) prelimbic, infralimbic (PrL-IL) and to a lesser extent to the (dorsal) medial prefrontal cortex (mPFC) via glutamatergic fibers.^11,12^ The interaction between the hippocampus and the prefrontal cortex is crucial for learning and memory and disturbances are likely linked to several psychiatric disorders, including schizophrenia.^13^

**Figure 1.**
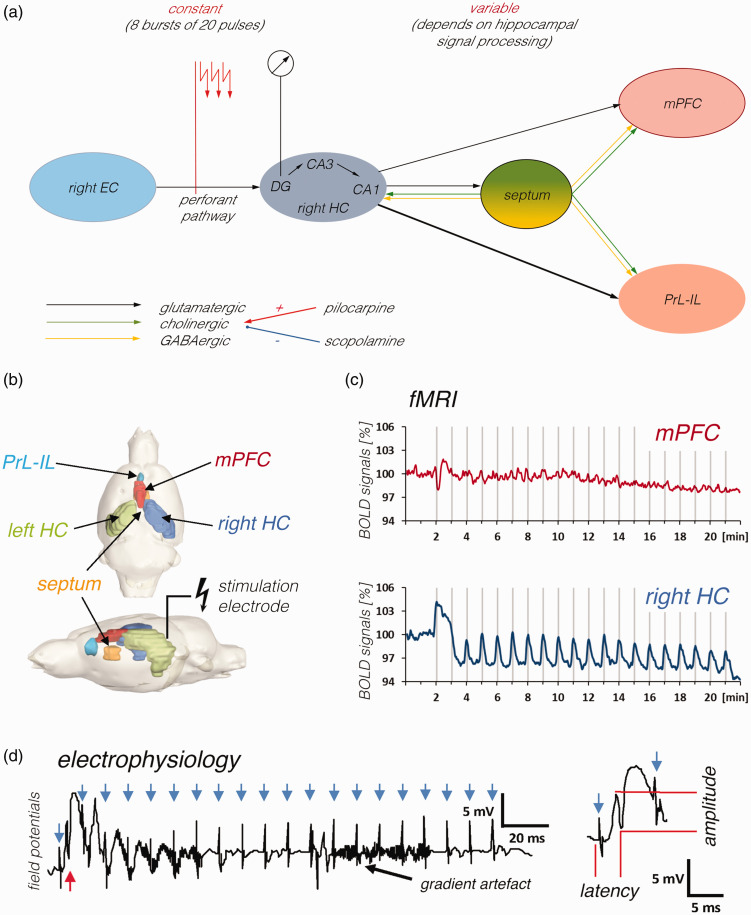
Experimental set up used for the simultaneous measurement of electrophysiological and fMRI data during electrical stimulation of the right PP. a: Scheme summarizing the main connections and brain areas that are activated by electrical perforant pathway stimulation. The right perforant pathway projects from the right entorhinal cortex (right EC) to the right dentate gyrus (DG), CA3 and CA1, thus electrical stimulation of this fiber bundle (indicated by the red arrows) will directly activate neurons in these regions (stimulus-induced neuronal responses were recorded from granule cells in the dentate gyrus, i.e., from neurons that only monosynaptically activated). In addition, activated DG granule cells project to the CA3 where pyramidal cells project to the CA1. Principal neurons in the CA1 (and subiculum) target neurons in the septum and the prefrontal cortex (dorsal part: medial prefrontal cortex – mPFC, ventral part: prelimbic, infralimbic cortex – PrL-IL). In addition, activated septal neurons project to the hippocampus as well as prefrontal cortex regions. Projections to the hippocampus and from the hippocampus are glutamatergic (black arrows) and the projections from the septum are mainly cholinergic (green arrows) and GABAergic (yellow arrows). Whereas the input activity to the dentate gyrus can be adjusted in all experiments to a constant value by the stimulation protocol, the input activity to the septum and prefrontal cortex depends on the efficacy of signal propagation through the hippocampus and is therefore variable. b: Location of the stimulation electrode and all analyzed VOIs. c: fMRI was used to measure BOLD signal changes in various brain regions during the stimulation. Gray vertical bars indicate the 8 s stimulation periods. d: Concurrent with fMRI signal measurement, extracellular field potentials were recorded in the right dentate gyrus during each pulse burst stimulation period (i.e., 20 pulses with an inter-pulse interval of 10 ms). Each pulse bursts stimulation elicited only one population spike after the first pulse (red arrow), thus during one stimulation period 8 population spikes were elicited. Blue arrows indicate the location of stimulation artifacts. Right side: Magnification of the first response. Population spike amplitude and latency were measured to monitor drug-related changes in neuronal responses.

Similar to the prefrontal cortex, fibers of perforant pathway (PP) as well as intrahippocampal connections (e.g., mossy fibers and Schaffer collaterals) are glutamatergic. Thus, in both the hippocampus and prefrontal cortex (i.e., PrL-IL and mPFC) an increase in local neuronal network activity is mainly controlled by glutamatergic inputs, whereas concurrently arriving cholinergic inputs modify this activity (Figure 1(a)). Given the important role of the cholinergic system in modulating many cognitive functions, we investigated whether it also affects BOLD signaling so that fMRI could be used to detect activation of the cholinergic system under in vivo conditions.

We used functional magnetic resonance imaging (fMRI) in combination with electrical PP stimulation to visualize the role of the cholinergic system on signal processing in the hippocampus and prefrontal cortex and the resulting changes in hippocampal–prefrontal interactions. fMRI measures hemodynamic parameters, such as blood oxygenation or blood flow/volume, which, in turn, are controlled by neuronal activity. Thus, stimulus-related changes of fMRI signals in a given region point to an altered neuronal activity in this region. Because stimulus-related changes of fMRI signals in the hippocampus mainly depend on the quality of neuronal signal processing,^14^ cholinergic modulation of neuronal network activity may become detectable by fMRI, i.e., fMRI responses to an identical stimulus in presence of a mACh agonist or antagonist should differ when the cholinergic system clearly modifies neuronal signal processing. There are already several studies showing that activation of mACh m3/5 receptors can lead to cerebral vasodilation,^15–19^ but so far only one study has been able to show an influence of locally injected mACh on BOLD signals in the primary visual cortex.^20^

To activate hippocampal neuronal circuits, we electrically stimulated the right PP that projects monosynaptically to the dentate gyrus, hippocampus proper (i.e., CA1–3) in the absence or presence of a mACh receptor agonist (pilocarpine) and/or antagonist (scopolamine). In this experimental setup the applied electrical pulses define an additional incoming activity to the hippocampus and this stimulus-related activity was kept identical for all experiments. The activity that eventually leaves the hippocampus and activates neurons in the septum or prefrontal cortex depends on the quality of signal processing in the hippocampus, i.e., it changes as soon as signal processing in the hippocampus is modified. As efferent fibers of the hippocampus also reach into the septum, activation of the endogenous cholinergic system also occurs, which in turn can modulate hippocampal signal processing (Figure 1(a)). It should be noted that most direct manipulations of neurons/fiber pathways by electrical stimulations elicit a neuronal activation pattern that does not correspond to the most physiological state. However, unlike external sensory stimulation (which elicits central neuronal activation in the most physiological state), direct electrical stimulations of a monosynaptically projecting central pathway are easily adjustable (in terms of time, duration, intensity, and pattern) so that it is possible to study exactly how a given parameter affects specific parameters such as the BOLD and/or CBV response.

Therefore, by comparing mACh-receptor agonist/antagonist-related changes in stimulus-induced BOLD responses in the hippocampus and prefrontal cortex, the effects of the cholinergic system on local neuronal circuit activity as well as brain wide neuronal circuit activity can be monitored. Furthermore, presence of mACh-receptor antagonists during stimulation should modify fMRI responses when PP stimulation effectively activates the endogenous cholinergic system.

## Material and methods

### Animals

Animals were cared for and used according to a protocol approved by the Animal Experiment and Ethics Committee and in conformity with European conventions for the protection of vertebrate animals used for experimental purposes as well as institutional guidelines 86/609/CEE (November 24, 1986). The experiments were approved by the animal care committee of Saxony-Anhalt state (No. 42502–2–1406 DZNE) and performed according to the Animal Research: Reporting *In Vivo* Experiments (ARRIVE) guidelines. Male Wistar Han rats (age 9–13 weeks) were housed individually under conditions of constant temperature (23°C) and maintained on a controlled 12 h light:12 h dark cycle. Food and tap water were provided *ad libitum*.

### Experimental design

This is an exploratory study. No prior information was available which would have enabled us to perform sample size estimations based on evidence. We thus chose sample sizes which are standard in the field (n > 8). Animals were excluded only in case of insufficient electrode position, i.e., as soon as stimulation with a test pulse triggered a population spike with an amplitude of less than 5 mV. In this way, a total of 64 animals could be included in the study (Table S1). Animals were randomly assigned to groups for different mACh modulator treatments.

### Cholinergic modulators

We purchased pilocarpine hydrochloride, scopolamine hydrobromide, and scopolamine methylbromide from Merck (Darmstadt, Germany). We dissolved all drugs in a sodium chloride solution (0.9%). We applied all drugs intraperitoneally at the following concentrations: pilocarpine (1 mg/kg), scopolamine (1 mg/kg) and methylscopolamine (1 mg/kg). Cholinergic antagonists were applied 25 min before the fMRI experiment started. To effectively reduce peripheral effects of pilocarpine on hemodynamic parameters, such as mean arterial blood pressure,^21^ this drug was always applied in combination with a cholinergic antagonist (methylscopolamine or scopolamine); i.e., in these experiments methylscopolamine was applied 25 min before the start of the fMRI measurement and pilocarpine 5 min later. Methylscopolamine (1 mg/kg) alone increased heart rate but had as described previously no effect on blood pressure, core temperature, and motor activity.^22^

### Surgical procedure

We implanted electrodes as previously described in detail.^23^ Briefly, we placed a bipolar stimulation electrode (114 µm in diameter, made from a Teflon-coated tungsten wire, impedance 18–20 KΩ) into the PP in the right hemisphere at the coordinates AP: −7.4, ML: 4.1 mm from Bregma, DV: 2.0 to 2.5 mm from the dural surface. We lowered a monopolar recording electrode (114 µm in diameter, made from a Teflon-coated tungsten wire) into the granule cell layer of the right dentate gyrus at AP: −4.0 mm, ML: 2.3 mm from Bregma, DV: 2.8 to 3.2 mm from the dural surface. We calculated all coordinates according to.^24^ We verified the correct placement of stimulation and recording electrodes during the implantation by measuring monosynaptic field potentials, and we adjusted the position of the electrodes according to the signal. We placed grounding and reference silver electrodes on the dura of the left side of the cranium. We fixed all electrodes with dental acrylic (Paladur, Hereaus, Germany) and anchored them on the skull with plastic screws. Following surgery, the animals were housed individually and given 1 week for recovery before experiments.

### Combined fMRI and electrophysiological measurements

We performed all experiments combining electrophysiology and fMRI with a 9.4 T Bruker Biospec 94/20 animal scanner, equipped with a BGA12 HP (440 mT/m) gradient system. We used a 72 mm volume coil (Bruker Biospin MRI GmbH, Ettlingen, Germany) for radio frequency (RF) excitation and a 20 mm planar surface coil (Bruker Biospin MRI GmbH) for signal reception. We initially acquired a B0-field map for local shimming using an ellipsoid volume of interest (VOI) that covered the entire brain. We performed BOLD-fMRI using a gradient echo planar imaging (EPI) sequence with the following parameters: repetition time (TR) 2000 ms, echo time (TE) 20.61 ms, flip angle: 90°, bandwidth: 326,087 Hz, slice thickness 0.4 mm, inter-slice distance 0.1 mm, field of view (FOV) 25.6 × 25.6 mm, and matrix 128 × 128 (in plane resolution 0.2 × 0.2 mm).

We initially anesthetized all animals with isoflurane (2%, in 50:50 N_2_:O_2_ [v:v]) and then fixed them into the head holder and connected them to stimulation and recording electrodes. Then, we reduced the isoflurane concentration to 1.5% and subcutaneously injected a bolus of medetomidine (50 µg/kg) (Dorbene, Pfizer GmbH). Ten minutes later, we reduced the isoflurane concentration to 0.4% and started continuous subcutaneous application of medetomidine (100 µg/kg). Five minutes later, we interrupted isoflurane application. We provided heating from the ventral side of the animal and monitored heart rate and breathing rate during the entire experiment (Life Monitoring Unit, Bruker). We did not detect significant differences in these parameters between all individual experimental groups.

We used bipolar pulses (pulse width 0.2 ms) to stimulate the PP; the intensity was always set to 250 µA. The stimulation protocol consisted of 20 stimulation trains given every minute after a 2-min baseline. Every stimulation train lasted 8 s and it was followed by 52 s without stimulation. Each stimulation train comprised eight pulse bursts, one burst per second. Each pulse burst comprised 20 pulses with an inter-pulse onset of 10 ms (100 Hz). When we measured animals more than once, we performed the subsequent measurement after a minimum of 7 days.

We filtered the electrophysiological responses (population spikes) with an antialiasing filter, i.e., cutoff frequencies below 1 Hz and above 5000 Hz, using an EX1 amplifier (Science Products, Hofheim, Germany), transformed by an analog–digital interface (power-CED, Cambridge Electronic Design, Cambridge, UK) and stored on a personal computer with a sampling rate of 5000 Hz. The low sampling rate did not affect the shape of the recorded population spikes because the underlying signal did not contain frequencies >1 kHz. Additionally, we removed 50 Hz noise using a HumBug noise eliminator (Quest Scientific, North Vancouver, BC, Canada). We also recorded in a separate group of animals outside the scanner local field potentials (LFP) to monitor ongoing neuronal activity in the dentate gyrus and prefrontal cortex during and after one stimulation period. For that a second recording electrodes was implanted in the infralimbic cortex (at the coordinates AP: +2.7 mm, ML: +0.5 mm from Bregma, DV: 3.1 mm from the dural surface). Signals were filtered (high pass filter: 0.1 Hz, low pass filter: 5000 Hz) using EX1 amplifiers (Science Products, Hofheim, Germany) and transformed by an analog–digital interface (power-CED, Cambridge Electronic Design, Cambridge, UK). Data were recorded using a sampling rate of 5000 Hz with Spike2 (version 6).

We used trigger pulses that were generated by the scanner at the beginning of every volume, i.e., every 2 s, to synchronize fMRI image acquisition and electrophysiological stimulations. Electrical stimulation started with a 270 ms delay to prevent an overlay of electrophysiological responses with scanner-induced artifacts. Each fMRI measurement started with an initial 2 min period without any stimulation (to determine baseline BOLD signals) before we applied the stimulation protocol.

After fMRI measurements, we obtained 20 horizontal anatomical spin-echo images (*T*_2_-weighted) using a rapid acquisition relaxation enhanced (RARE) sequence ^25^ with the following parameters: TR 3000 ms, TE_effective_ 33 ms, bandwidth: 33,333 Hz, slice thickness 0.4 mm, FOV 25.6 × 25.6 mm, matrix 256 × 256, RARE factor 8, and averages 3. The total scanning time was 4 min 48 s. The slice geometry, i.e., 20 horizontal slices, was identical to the previously obtained gradient-echo EPI.

### Data processing and analysis

We loaded the functional data and converted it to BrainVoyager data format. We applied a standard sequence of pre-processing steps implemented in the BrainVoyager QX 2.8.0 software (Brain Innovation, Maastricht, the Netherlands). i.e., slice scan time correction, three-dimensional (3 D) motion correction (trilinear interpolation and reduced data using the first volume as a reference), and temporal filtering (full width at half maximum [FWHM] using three data points) to each dataset. We did not apply spatial smoothing or distortion correction tools.

### VOI analysis

We aligned each individual functional imaging dataset to a 3D standard rat brain using the 3D volume tool implemented in BrainVoyager QX 2.8.0. Coregistration was manually performed using anatomical landmarks. We marked the following VOIs in the 3D standard rat brain: right/left dorsal hippocampus, medial prefrontal cortex region, prelimbic and infralimbic cortices, and septum. We then calculated the averaged BOLD time series of all voxels located in one VOI for each individual animal using the VOI analysis tool implemented in BrainVoyager QX 2.8.0. Each individual BOLD time series was normalized using the averaged BOLD signal intensity in the first two minutes as 100%. We then averaged normalized BOLD time series and depicted them as mean BOLD time series ± standard deviation (SD).

We calculated significant changes in baseline BOLD signals as described previously using paired *t*-tests (Angenstein, 2019). For each animal, we determined the lowest BOLD signal intensity measured between frames 3 and 58 (i.e., before onset of stimulation). We performed then for each subsequent time point (i.e., from frame 60 to 660) a two-sample equal variance *t*-test with Bonferroni correction. We considered differences to be significant when p < 0.01.

To identify stimulus-induced changes of BOLD signals (i.e., BOLD response) we calculated event-related BOLD responses. For that signal intensities starting six frames before stimulus onset (-12 s until 0 s), during stimulus presentation (between 0 and 8 s, which corresponds to four frames) and the following 15 frames (8 to 38 s) after the end of the stimulus. To avoid the confounding effect of putative variations in baseline BOLD signal intensities (i.e., baseline BOLD shifts) on the calculated BOLD response each BOLD response was related to BOLD signal intensities of the stimulus over the preceding 12 s, which were set to 100%.

To visualize how different brain regions were affected in their activity by cholinergic modulation, we compared differences in BOLD responses between two conditions (e.g., control vs scopolamine) using an unpaired *t*-test. We considered differences to be significant when p < 0.01.

### General linear model (GLM) analysis

We used each individual functional dataset for a multiple-subject GLM (analysis implemented in BrainVoyager QX 2.8.0. We analyzed functional activation using the correlation of the observed BOLD signal intensity changes in each voxel with a predictor (hemodynamic response function) generated from the given stimulus protocol (see above). Based on this, the appropriate 3D activation map could be generated. To calculate the predictor, the square wave representing stimulus on and off conditions was convolved with a canonical double gamma hemodynamic response function (onset 0 s, time to response peak 5 s, and time to undershoot peak 15 s). To exclude false-positive voxels, we considered only those with a significance level (p value) above the threshold set by calculating the false discovery rate (FDR) with a q value of 0.001 (which corresponds to a *t*-value greater than 3.8 or p < 1.2 × 10^−4^).

### Determination of changes in local CBV

We measured CBV changes with identical imaging parameters but 5 min after intravenous injection (through the tail vein) of 20 mg/kg ultrasmall superparamagnetic iron-oxide (USPIO) nanoparticles (Molday ION, BioPAL Inc.™, Worcester, MA, USA). We chose that concentration because it is close to the optimal contrast-to-noise ratio and has a low (calculated) BOLD contribution to the CBV-weighted MRI.^26^

To determine the relationship between CBV and BOLD signals, a Pearson Correlation test was performed using the free statistical software for bivariate descriptive statistics.^27^

### Analysis of electrophysiological data

We measured the amplitude of the population spike in mV (from the first most positive point to the following most negative point) and the latency in ms (from the onset of the stimulus artifact to the most negative point, Figure 1(d)). All absolute measurements were averaged and depicted as the arithmetic mean ± SD. To visualize how the population spike amplitude and latency were affected by cholinergic modulation, we average the responses in one train and compared them between two conditions (e.g., control vs scopolamine) using a Student’s unpaired *t*-test. We considered differences to be significant when p < 0.05. All spectrograms were computed using Spike2 (version 6) using a Hanning window (4096 block size).

## Results

### Variation in BOLD signals in the absence or presence of a mACh receptor agonist or antagonist

**Figure 2.**
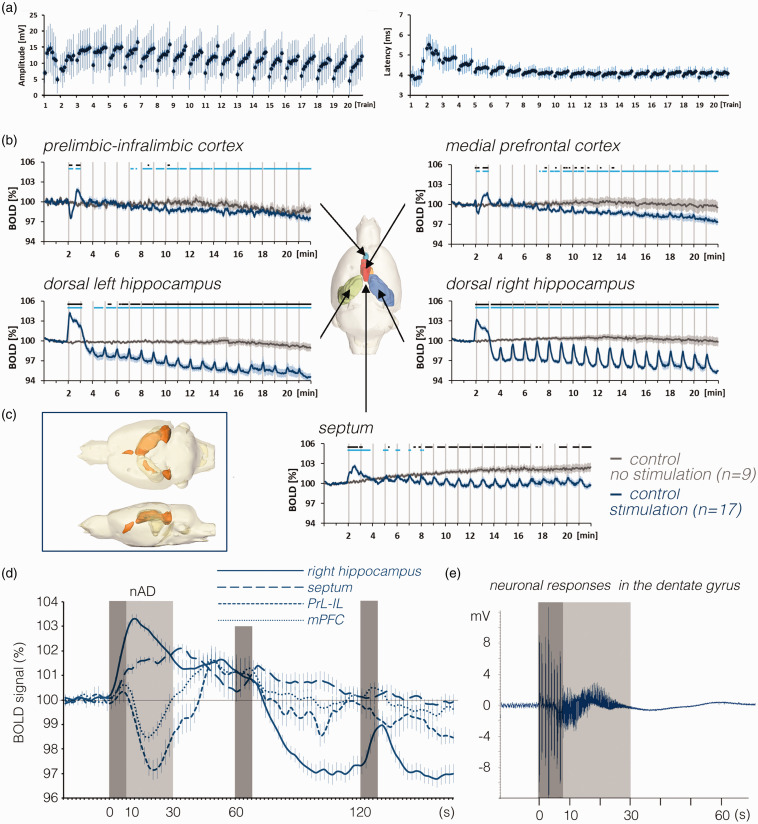
BOLD responses in the brain and neuronal responses in the right dentate gyrus during repetitive stimulations of the right PP. a: Neuronal responses during all individual stimulation trains in the right dentate gyrus (i.e., 8 population spikes (1 per burst) per train). After the first stimulation train, population spike latencies temporarily increased and normalized after the third stimulation period. In addition, after the third stimulation period, there was a consistent response pattern, i.e., similar to a continuous increase of responses in each individual stimulation train. b: Mean BOLD time series in various regions during stimulation of the right perforant pathway (blue lines) compared with the unstimulated condition (dark gray lines). Gray vertical bars indicate the 8 s stimulation periods. Note the baseline BOLD signal shift in the right and left hippocampus during the stimulation experiment, whereas in the septum during repetitive stimulations, baseline BOLD signals remained almost stable but increased when no stimulations were applied (significant differences from baseline BOLD signals are indicated as a light blue line at the top of each graph). Periods of significant differences in BOLD signals between the two conditions are depicted as a black line at the top of each graph. c: Spatial distribution of significantly activated voxels in the rat brain. d: Comparison of mean BOLD signal changes in the dorsal right hippocampus (solid line), septum (dashed line), mPFC (dotted line) and PrL-IL (light dotted line) during the first three stimulation periods. Dark gray boxes indicate periods of stimulation and the light gray box indicates the heavy h-nAD period. In the mPFC and PrL-IL BOLD signals tend to increase during the initial stimulation period whereas the delayed apparent negative BOLD response coincides with the h-nAD period. e: Simultaneously recorded field potentials in the dentate gyrus showed the presence of heavy spiking activity after the termination of the first perforant pathway stimulation period (indicated by the dark gray box). The period of heavy spiking activity (h-nAD, indicated by the light gray box) typically ended after 20–25 s.

First, we measured the development of BOLD signals under control conditions, i.e., when only medetomidine as sedative was present. Under this condition, variation in BOLD signals should reflect scanner-, analysis and/or sedation-related artifacts. Under control conditions, BOLD signals remained stable in all analyzed brain regions for 22 min, except the septum (Figure 2, S1), where there was a significant increase in BOLD signals at the end of the experiment.

Second, we repeated the same fMRI experiment in the presence of methylscopolamine or scopolamine. When one of these antagonists was present, the BOLD signals developed as observed in the control condition (Figure S1B).

Third, we reran the experiment in the presence of the mACh receptor agonist pilocarpine. Pilocarpine caused a significant decline in baseline BOLD signals in the PrL-IL, mPFC, and left and right dorsal hippocampus and attenuated the slow increase in BOLD signals that occurred in the septum in the control condition (Figure S2). When we administered pilocarpine together with scopolamine, the gradual decrease of the BOLD signal from baseline was still present however in the left and right dorsal hippocampus the decrease was no longer significant (Figure S2).

### Variation in BOLD signals during electrical stimulation of the PP in the absence or presence of a mACh receptor agonist or antagonist

In a second set of experiments, we electrically stimulated the PP and examined how the same mACh receptor modulators affect the stimulus-induced BOLD response.

### Stimulation of the PP in the absence or presence of methylscopolamine

Concomitant with the BOLD signal acquisition, we always recorded during stimulations the elicited extracellular field potentials in the right dentate gyrus. Except for the second stimulation train in which there was higher population spike latencies and smaller population spike amplitudes, all other trains showed a similar development of population spikes amplitudes and latencies (Figure 2(a)).

Under control condition, i.e., stimulation of the PP in the absence of any drugs, each stimulation train caused a positive BOLD response in the right dorsal hippocampus (Figure 2(b) and (d)). The first stimulation train triggered, in 17 out of 19 animals, a prolonged BOLD response that lasted until the beginning of the second stimulation train; thus, the second BOLD response was scarcely detectable. As previously observed (Angenstein, 2019; Helbing et al., 2013), this prolonged increase reflects the presence of hippocampal neuronal afterdischarges (h-nAD) that were only induced by the first stimulation train (Figure 2(e)). After the second stimulation train, baseline BOLD signals declined to a level that was significantly lower than before the stimulation started (Figure 2(d)). This reduced baseline BOLD signal level remained present until the end of the experiment. Nevertheless, all subsequent stimulation trains (trains 3 to 20) still triggered significant positive BOLD responses. That is, each stimulation train caused a significant increase in BOLD signals compared with the baseline BOLD signals currently present at each time point.

Stimulation of the right PP also affected BOLD signals outside the right hippocampal formation. In the left dorsal hippocampus, the decrease in baseline BOLD signals after the first period of stimulation was similar to that in the right dorsal hippocampus, but the positive BOLD responses during each stimulation train were significantly lower (Figure S3). In the septum, there was an initial prolonged increased BOLD response that was also followed by small positive BOLD responses during all subsequent stimulus trains. However, in contrast to the left and right dorsal hippocampus, there was not a significant decline of baseline BOLD signals. In the prefrontal cortex we observed an initial drop of the BOLD signal after the first stimulation train that was followed first by a short increase and then by a slow decrease lasting until the end of the recording (Figure 2(b)). Comparing the first BOLD response in in various regions revealed that the onset of the negative BOLD response in the prefrontal cortex was delayed by 8–10 s relative to the onset of the positive BOLD response in the dorsal right hippocampus. Thus, the negative response in the prefrontal cortex coincided with the presence of stimulus-induced h-nAD rather than to pulse related neuronal responses in the hippocampus (Figure 2(d) and (e)). This outcome agrees with the finding that all subsequent stimulation trains did not cause similar negative BOLD responses or h-nAD (Figure 2(b)).

**Figure 3.**
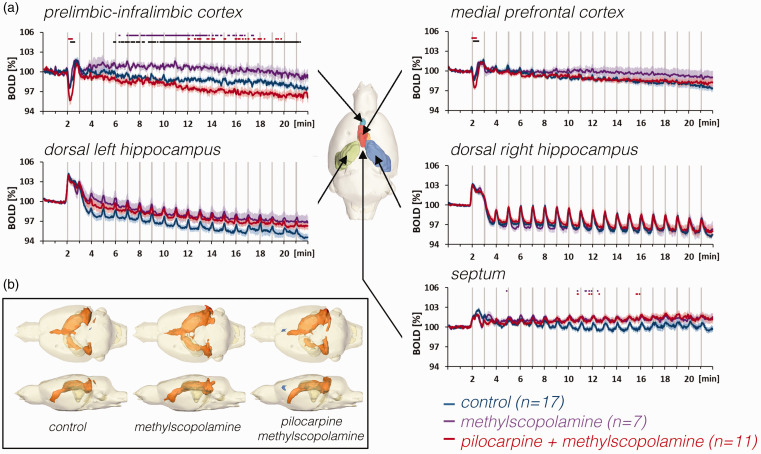
The effect of methylscopolamine and pilocarpine on BOLD responses in the hippocampus, septum, and prefrontal cortex during repetitive stimulations of the right perforant pathway. a: Methylscopolamine (purple lines), which was used to prevent peripheral effects of pilocarpine, already affected the development of baseline BOLD signals in the prelimbic-infralimbic cortex. When pilocarpine was applied (in the presence of methylscopolamine, red lines), baseline BOLD signals declined in the prelimbic-infralimbic cortex and the initial negative response in the prelimbic cortex, i.e., PrL-IL and mPFC) was significantly enhanced. By contrast, there were no significant effects in the right and left hippocampus and septum. b: Stimulation of the PP in the presence of methylscopolamine or pilocarpine (and methylscopolamine) caused a similar spatial distribution of significantly activated voxels in the rat brain.

Presence of methylscopolamine during stimulation had no effect on stimulus induced neuronal (Figure S4A) and BOLD responses, however, in the PrL-IL, methylscopolamine attenuated the decline of baseline BOLD signals below the initial level (Figure 3, purple lines). That is, although methylscopolamine did not affect BOLD baseline signals under resting conditions (Figure S1B), methylscopolamine altered BOLD baseline signals in PrL-IL during PP stimulation. Because methylscopolamine should not penetrate the blood-brain barriers, the effect of methylscopolamine should only act on luminal vascular components, such as the endothelium. Hence, it appears that neuronal activity is required to initiate a vascular response, which then was further modulated by intraluminal endothelial mACh receptors.

### Neuronal, BOLD and CBV responses after the first stimulation train

**Figure 4.**
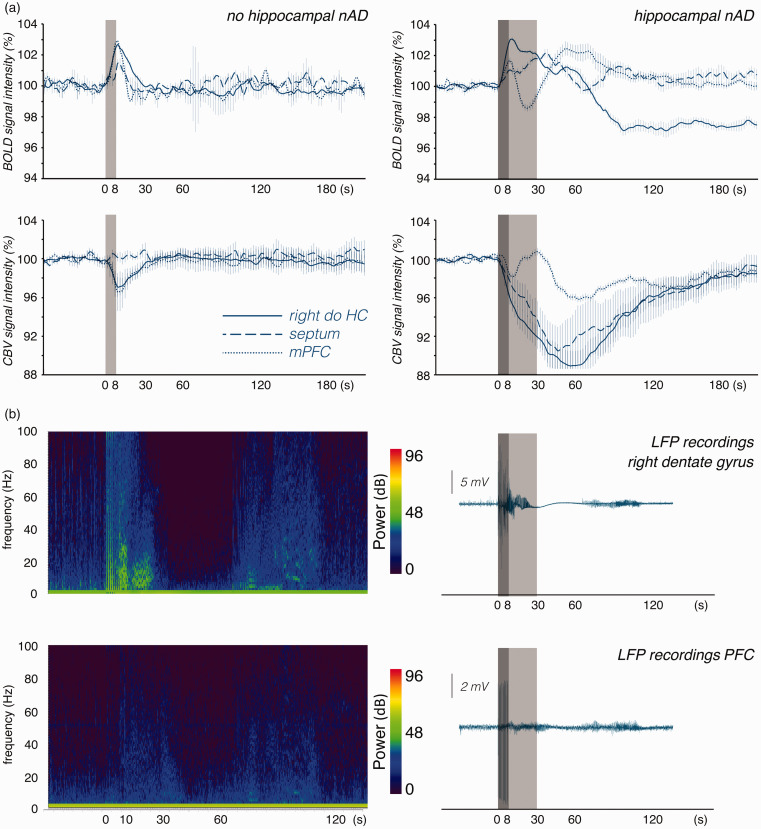
Development of BOLD and CBV signals in the right dorsal hippocampus (right do HC, blue lines), septum (blue dashed lines) and medial prefrontal cortex (mPFC, blue dotted lines) during a single stimulation period. a: *Left side*: When perforant pathway stimulation did not elicit h-nAD than a short positive BOLD response was generated in all three regions. The transient positive BOLD response was accompanied with a corresponding transient hyperemia (i.e., increase in CBV) in the right dorsal hippocampus and mPFC but not in the septum. (Note, that an increase in CBV relates to a decrease in the fMRI signal). *Right side*: When perforant pathway stimulation elicits h-nAD than BOLD signals develop differently in the three regions. During the period h-nAD BOLD signals only slowly declined in right dorsal hippocampus, increased in the septum and declined below the initial value in the mPFC. After h-nAD ceased BOLD signals declined below the initial value in the right dorsal hippocampus, slowly returned to the initial value in the septum. In the mPFC BOLD signals transiently increased and returned then to the initial value. During the period of h-nAD CBV strongly increased in the dorsal hippocampus and septum but declined in the mPFC. Thus, whereas h-nAD caused a functional hyperemia in the dorsal hippocampus and septum the blood supply was reduced in the mPFC. When h-nAD terminated then CBV increased again before it returned slowly to the initial value. b: Simultaneous electrophysiological recordings in the right dorsal hippocampus (upper part) and prefrontal cortex (lower part) during and after a single stimulation period. Left side: frequency spectrum. During stimulation (dark gray box), synchronized activity was observed in the right dorsal hippocampus and to a lesser extent in the prefrontal cortex. Synchronized activity increased in the prefrontal cortex during h-nAD (light gray box) and again 60 s later. The power values of the frequencies are given in decibels (dB). Right panel: Simultaneous LFP recordings in the right dorsal hippocampus and prefrontal cortex during a stimulation period (indicated by the dark gray box).

Because we observed the largest effects in the prefrontal cortex after the first measured stimulation period, we conducted a separate experiment to examine how the first stimulation period alters neuronal activities and associated CBV and BOLD signals. Again, stimulation triggered h-nAD in 10 out of 14 animals (CBV) or 10 out of 13 animals (BOLD). When stimulation did not induce h-nAD we observed *short positive BOLD responses* in the dorsal hippocampus (102.6 ± 0.3%) and prefrontal cortex (102.9 ± 0.6%) but to a less extend in the septum (101.4 ± 0.2%). We also detected a concurrent short functional hyperemia in these two regions (dorsal hippocampus: 97.1 ± 1.0%; prefrontal cortex:96.6 ± 1.1%) but not in the septum (99.8 ± 0.3%; Figure 4(a), left side). Thus, in the two regions an increase in BOLD signals relates to a functional hyperemia.

When the same stimulation also caused h-nAD, BOLD signals in the hippocampus initially increased during stimulation and slowly declined during the h-nAD period. By contrast, CBV increased during stimulation and further increased during the h-nAD period. In the septum, we observed a similar development of BOLD signals and CBV during the stimulation and the h-nAD period, i.e., a slight increase during the stimulation and a further increase during the subsequent h-nAD period. In contrast, in the prefrontal cortex, CBV increased during the stimulation but decreased during the h-nAD period and increased again after h-nAD terminated (Figure 4(a), right side). Thus, in the prefrontal cortex, the initial small increase in BOLD signals coincided with a concurrent transient hyperemia and the subsequent negative BOLD response corresponded with a normalization of blood volume, i.e., during the h-nAD period, there was no hyperemia in the prefrontal cortex. Only when h-nAD ceased did the blood volume increase a second time in the prefrontal cortex; this phenomenon again coincided with an increase in BOLD signals. Correlation of BOLD and CBV signal changes showed a close relationship between these two parameters (Table S2). This suggests that changes in BOLD signals depend primarily on concurrent changes in CBV.

In a separate group of animals, we simultaneously measured ongoing neuronal activity in the right dentate gyrus and prefrontal cortex during and after one stimulation period. As described above, the stimulation elicited neuronal afterdischarges in the hippocampus that lasted for approximately 15–20 s. The increased neuronal activity was followed by a brief period of approximately 30 s during which almost no neuronal activity was detected (Figure 4(b)). Simultaneously recorded signals in the prefrontal cortex showed increased neuronal activity during h-nAD, which was not followed by reduced activity. In both regions, neuronal activity transiently increased again after approximately 60 s (Figure 4(b)). Thus, in the two regions, BOLD and CBV signals did not follow stimulus-induced changes in ongoing neuronal activity.

### Stimulation of the PP in the presence of pilocarpine

**Figure 5.**
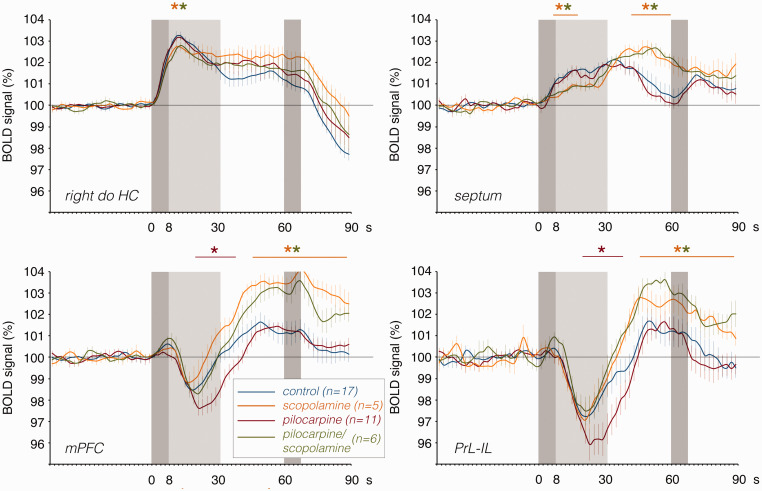
The effects of pilocarpine (red lines), scopolamine (yellow lines) or pilocarpine/scopolamine (green lines) on the first BOLD response in the dorsal right hippocampus, septum, mPFC, and PrL-IL when compared identical stimulation without drug treatment (blue lines). Whereas pilocarpine significantly enhanced the *negative BOLD response* in the prefrontal cortex, scopolamine reduced the BOLD response in the dorsal right hippocampus, increased the delayed positive component in the prefrontal cortex, and modified the hemodynamic response in the septum. The effect of pilocarpine on the negative BOLD response in the mPFC and PrL-IL was completely blocked by scopolamine. The dark gray box depicts the period of stimulation and the light gray box the time period of heavy h-nAD.

In the presence of pilocarpine, repetitive PP stimulation caused similar BOLD responses in the right, left hippocampus and septum as observed during control conditions (Figures 4(a) and 5). By contrast, pilocarpine significantly enhanced the negative BOLD response in the PrL-IL and mPFC after the first stimulation train (Figures 3(a) and 5). Furthermore, in the presence of pilocarpine, baseline BOLD signals again declined during repetitive stimulations in the PrL-IL; thus, the effect of methylscopolamine on the decline in BOLD signals in the PrL-IL was completely reversed by pilocarpine (Figure 3(a)). Field potential recording in the dentate gyrus, however, did not reveal a significant effect of pilocarpine on stimulus-induced neuronal responses (Figure S4B).

### Stimulation of the PP in the presence of scopolamine

**Figure 6.**
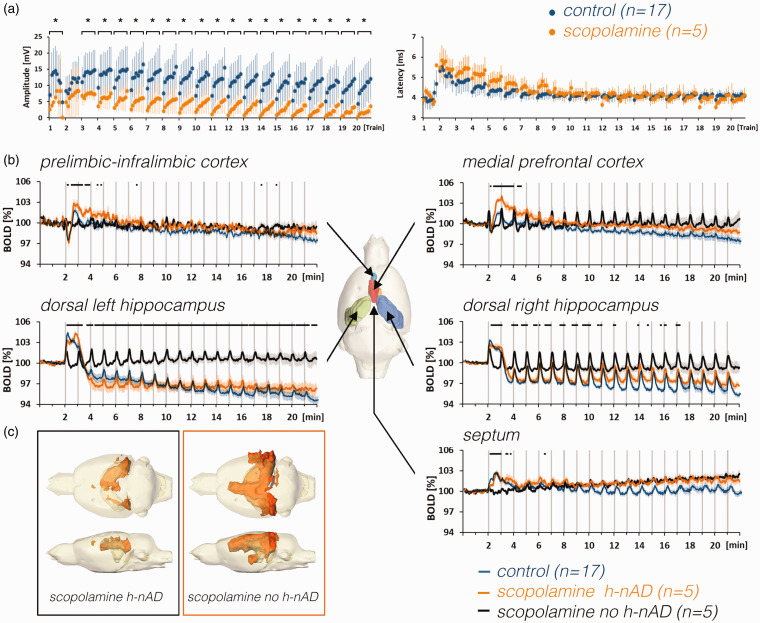
Effect of scopolamine on stimulus-induced neuronal responses in the dentate gyrus and BOLD responses in the rat brain. a: Scopolamine attenuated stimulus-induced neuronal response in the right dentate gyrus (asterisks indicate significant differences to control group; p < 0.05). In contrast to the population spike amplitude, scopolamine did not alter the population spike latency. b: Mean BOLD time series of scopolamine-treated animals that either elicited h-nAD (orange lines) after the first stimulation period or did not elicit h-nAD (black lines) in comparison to untreated animals that elicited h-nAD (control, blue lines). The presence of h-nAD coincided with reduced baseline BOLD signals in the left and right hippocampus, the presence of a negative BOLD response in the prefrontal cortex, the presence of an initial positive BOLD response in the septum, and suppression of positive BOLD responses in the medial prefrontal cortex. c: The spatial distribution of significantly activated voxels in the rat brain of animals that either generated after the initial stimulation period in the presence of scopolamine with or without h-nAD.

In the presence of scopolamine, the first stimulation train only caused h-nAD in half of the animals (5 out of 10 animals); therefore, we divided the group of scopolamine-treated animals into two groups: one group with and one group without h-nAD. The presence of h-nAD caused, as described above, a short-lasting negative BOLD response in the prefrontal cortex that was absent when PP stimulation did not trigger h-nAD. When no h-nAD were triggered, then the first and all subsequent stimulation trains only caused clear positive BOLD responses in the prefrontal cortex (Figure 6(b), S5).

In scopolamine-treated animals that had h-nAD, BOLD time series in the right and left hippocampus were similar compared with untreated animals, except that the initial BOLD response was significantly attenuated (Figures 5 and 6(b)). In the mPFC and PrL-IL, BOLD signals significantly increased after the initial decline, whereas the initial short-lasting negative BOLD response was not significantly altered by scopolamine. In the septum, scopolamine reduced the initial part of the BOLD response and increased the subsequent part of the response; thus, in the septum an altered hemodynamic response was observed (Figure 5).

Field potential recordings in the dentate gyrus showed that population spike amplitudes were significantly reduced in scopolamine-treated animals (Figure 6(a)). Thus, scopolamine affected signal processing in the dentate gyrus.

### Stimulation of the PP in the presence of pilocarpine and scopolamine

**Figure 7.**
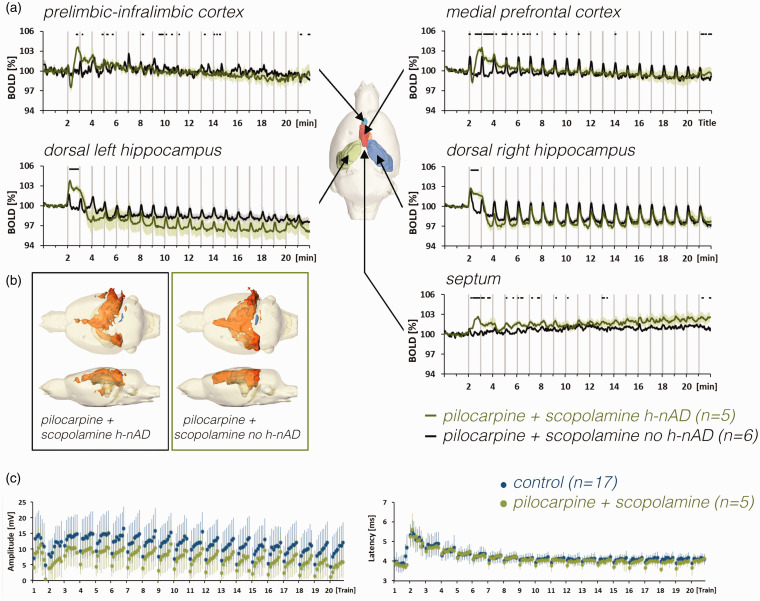
a: Comparison of BOLD time series of pilocarpine- and scopolamine-treated animals that either elicited h-nAD after the first stimulation period or did not elicit h-nAD. b: The spatial distribution of significantly activated voxels in the brain of animals with or without h-nAD after the initial stimulation period in the presence of pilocarpine and scopolamine. c: Summary of electrophysiological responses measured in the right dentate gyrus during control conditions (blue dots) and in presence of pilocarpine and scopolamine.

Co-application of the two drugs before repetitive stimulations again affected the incidence of h-nAD after the first stimulation period. Specifically, in only about half of the animals (5 out of 11 animals), the first PP stimulation period triggered h-nAD (Figure 7(a), S6).

In general, BOLD time series observed after the co-application of pilocarpine and scopolamine were similar to the ones observed during application of scopolamine alone, i.e., the specific effect of pilocarpine on the initial negative BOLD response in the PrL-IL and mPFC was blocked by scopolamine, whereas the specific effects of scopolamine (i.e., the transient increase in BOLD signals in the mPFC after the first stimulation train) persisted (Figure 5 green lines).

## Discussion

In the current study, we electrically stimulated the right PP with consecutive trains of high-frequency pulse bursts and measured, in the presence of a mACh receptor agonist/antagonist, BOLD signal changes in the hippocampal formation and in some of its target regions (i.e., septum, mPFC, and PrL-IL) as well as neuronal responses in the right dentate gyrus. Based on electrophysiological recordings, stimulation of the PP triggered two basic forms of neuronal responses in the dentate gyrus. First, granule cells responded to each pulse and subsequently generated neuronal afterdischarges (h-nAD) that last approximately 15–20 s (usually train 1). Second, granule cells only responded to the applied pulses but did not generate h-nAD (usually train 2–20 or in the presence of scopolamine). Based on measured BOLD signals, electrical PP stimulation caused four different hemodynamic responses: first, an immediate transient increase in BOLD signals with fast normalization to the initial value (*short positive BOLD response*); second, an immediate transient increase with delayed normalization (*long positive BOLD response*); third, a delayed (by about 8–10 s) transient decline in BOLD signals with normalization (*negative BOLD response*); and fourth, a sustained decline in BOLD signals without normalization to the initial value during the following 20 min (*negative baseline BOLD shift*).

Comparing BOLD signal changes and concurrently recorded neuronal responses in the dentate gyrus revealed that *short positive BOLD responses*, which we mainly observed in the hippocampus and occasionally in the mPFC, were triggered by pulse-related neuronal responses, whereas *long positive BOLD responses*, which we observed in the hippocampus as well as in the septum, were only triggered when h-nAD were present (Figure 3). Only when there were *long positive BOLD responses* in the hippocampus/septum were there *negative BOLD responses* in the prelimbic cortex; thus, the presence of h-nAD triggered *negative BOLD responses* in the prefrontal cortex. By contrast, there was a *negative baseline BOLD shift*, which we detected in the hippocampus and prelimbic cortex but not in the septum, after repetitive stimulations, after one stimulation period that induced h-nAD, as well as after continuous activation of mACh receptors by pilocarpine.

In general, activation of mACh receptors by pilocarpine during PP stimulation enhanced the negative BOLD response in both prefrontal cortex regions, i.e., an activated cholinergic system affected only BOLD responses generated outside the directly activated hippocampal formation. In contrast, inhibition of mACh receptors by scopolamine reduced the initial positive BOLD response and neuronal responses in the right dorsal hippocampus and increased a late positive BOLD response component in the prefrontal cortex and septum. From these observations, we conclude that: (I) stimulation of the PP with high-frequency pulse bursts activates the endogenous cholinergic system (hence the effect of scopolamine) and (II) the cholinergic system modifies stimulus-induced fMRI BOLD responses mainly indirectly, i.e., via influencing signal processing in local neuronal circuits

### The effect of nAD on subsequent BOLD signal changes

In contrast to short low frequency pulse stimulations high-frequency pulse burst stimulation elicited h-nAD that correspond to ictal activity, as described previously by Bragin and colleagues.^28^ When PP stimulation induced h-nAD, we observed two main effects, first, a *long positive BOLD response* in the hippocampus and septum; and second, *a negative BOLD response* in the prefrontal cortex.

A negative BOLD response in the prefrontal cortex during h-nAD indicate a temporarily reduced blood oxygenation, which, in turn, could be the result of higher oxygen consumption (as a result of higher neuronal activity), reduced blood supply (as a result of vasoconstriction), or the combination of both. Measurement of blood volume changes during stimulation indicated that h-nAD resulted, as expected, in an immediate functional hyperemia in the hippocampus and septum but, unexpectedly, not in the mPFC. There, an initially developing hyperemia was abolished during the h-nAD and only reappeared when h-nAD ceased.

Based on the development of the BOLD and CBV signals (Figure 3(a)), we hypothesize that during PP stimulation, the initial pulse-related neuronal activity in the hippocampus was propagated to the prefrontal cortex and produced a corresponding functional hyperemia, probably via glutamatergic transmission. However, once h-nAD have been generated, the neuronal activity now arriving in the prefrontal cortex counteracts the functional hyperemia that develops there. Electrophysiological recordings showed that during h-nAD, neuronal activity was also increased in the prefrontal cortex (Figure 4(b)). This indicates that, in addition to the glutamatergic transmission, other transmitter systems became activated during hippocampal nAD.

Hippocampal efferent fibers project to the prefrontal cortex and (medial) septum, which in turn projects via cholinergic and GABAergic fibers back to the hippocampus and to the prefrontal cortex.^10,29^ BOLD and especially CBV signals increased significantly in the septum when h-nAD were generated but remained almost unchanged when h-nAD were absent. Thus, according to BOLD signals, h-nAD elicited significantly stronger neuronal activity in the septum than during the initial period of stimulus-induced hippocampal activity. Only after h-nAD terminated did hyperemia reappear in the prefrontal cortex, i.e., factors that neutralize glutamatergic-mediated hyperemia vanished. Thus, based on BOLD signal changes, we conclude that, in particular, efferent activity from the septum (i.e., cholinergic and/or GABAergic) modify BOLD signals in the mPFC during h-nAD.

### The role of mACh receptors in BOLD signal changes

It has been convincingly demonstrated that direct activation of vascular mACh3 and mACh5 receptors, e.g., by cholinergic terminals, causes vasodilation and, consequently, hyperemia.^15,30,31^ However, the actual impact of an activated or inhibited cholinergic system on measured BOLD responses is not completely understood because the influence of cholinergic-mediated mechanisms on vascular responses are normally superimposed by concurrently acting glutamatergic-mediated neurovascular coupling mechanisms, because glutamate is the main excitatory transmitter for central signal processing. So far the role of an activated cholinergic system on BOLD signals has been studied by local ^20^ or, similar to the current study, by systemic application of a cholinergic agonists.^21^ In those studies, the observed effects critically depended on local concentrations of the applied drug and their elimination rate. For example, there were different effects of injected ACh on stimulus-induced BOLD signal changes near and far away the injection site.^20^ In our study, modulators of mACh receptors were always applied before the fMRI experiment, that is, 20 minutes (pilocarpine) or 25 minutes (scopolamine) before the first stimulation period, so we did not observe the direct effects of these drugs on the vasculature but rather their modulatory effects on subsequent neurovascular coupling processes.

In our study, continuous mACh receptor activation by pilocarpine had two main effects on BOLD signals: first, a slowly developing decline of baseline BOLD signals in the hippocampus and prefrontal cortex during the resting condition; and second, a significant enhancement of the negative BOLD response in the prefrontal cortex during PP stimulation.

In general, a shift of baseline BOLD signals in an fMRI dataset could be the result of scanner-, data analysis-, and/or sedation-related artifacts.^32–34^ Given that we only observed a significant decline in baseline BOLD signals in the presence of pilocarpine but not in untreated or antagonist-treated animals, we conclude that mACh receptor-mediated mechanisms affect the development of baseline BOLD signals. This finding would support previous observations that baseline BOLD shifts also result from neurophysiological processes.^35,36^

The effect of pilocarpine was more specific on the formation of the *negative BOLD response* in the prefrontal cortex during PP stimulation. As mentioned above, the delayed *negative BOLD response* is a result of a temporal dissociation between h-nAD-induced neuronal activity and vascular response, so that increased neuronal activity was not accompanied with concurrent but delayed hyperemia, which in turn then caused the observed transient reduced blood oxygenation. Thus, the enhanced *negative BOLD response* in the prefrontal cortex in the presence of pilocarpine likely reflects an altered neuronal activity in these regions as a result of mACh receptor activation. There are three feasible mechanisms that may alter neuronal activity in the prefrontal cortex: first, a mACh-receptor-mediated modification of h-nAD followed by altered efferent hippocampal activity to the mPFC; second, a mACh-receptor-activation-mediated change in efferent septal neuron activity, which in turn modify neuronal activity in the prefrontal cortex; and third, altered susceptibility of prefrontal neurons toward incoming activity (from the hippocampus and/or septum). According to fMRI data, pilocarpine did not significantly modify BOLD responses in the hippocampus and septum (Figure 5); it also did not alter electrophysiological responses in the dentate gyrus. Thus, mACh-receptor-mediated effects on neurons in the prefrontal cortex are more likely the cause for the enhanced negative BOLD response. In contrast to the negative part of the BOLD response in the prefrontal cortex, the subsequent positive component, which relates to the delayed increase in blood volume, did not change. This finding indicates that although the negative and subsequent positive component were the result of h-nAD they did not necessarily reflect a single neuronal mechanism, but rather two related mechanisms, in which only the first component was affected when the cholinergic system was already activated before PP stimulations.

To confirm the specific involvement of mACh receptors in mediating the enhanced negative BOLD response in the prefrontal cortex, we co-applied scopolamine with pilocarpine. Application of scopolamine before pilocarpine normalized but did not eliminate the *negative BOLD response.* Although the cholinergic system can modulate the *negative BOLD response* in the prefrontal cortex, it was not causative for it. When we co-applied scopolamine and pilocarpine, the subsequent positive component of the BOLD response in the prefrontal cortex significantly increased. This again indicates that the negative component and the late positive component of the BOLD response in the prefrontal cortex reflect two different neuronal mechanisms.

Furthermore, scopolamine applied alone before the stimulation also increased the delayed positive BOLD component and did not attenuate the *negative BOLD response* in the prefrontal cortex; hence, only the delayed positive component is sensitive to mACh receptor inhibition. The observation that scopolamine alone already affected the late positive component in the prefrontal cortex during stimulation confirmed that the mACh system was active. In contrast to pilocarpine, scopolamine (alone or in the presence of pilocarpine) also affected the BOLD response in the septum; in particular, the initial BOLD signal increase was attenuated, whereas a later component was increased. This altered shape of the BOLD response in the septum should reflect an altered neuronal activation pattern of septal neurons, which in turn should also result in an altered pattern of efferent activity to the prefrontal cortex.

Based on these results, we now assume the following. (i) Stimulation of the PP directly and/or via the hippocampal trisynaptic circuit activates projection neurons in the hippocampus (i.e., CA1 and subiculum), which in turn activate neurons in the prefrontal cortex via glutamatergic fibers and elicit *short positive BOLD responses* (i.e., functional hyperemia) there. Simultaneous activations of septal neurons are not sufficient to elicit detectable hemodynamic responses. (ii) Once stimulation triggers h-nAD, septal neurons are massively activated; thus, neurons of the prefrontal cortex receive increased cholinergic and GABAergic inputs from the septum in addition to increased hippocampal (glutamatergic) inputs. (iii) While hippocampal glutamatergic inputs still trigger functional hyperemia in prefrontal cortex, slightly delayed additional septal inputs counteract this functional hyperemia, ultimately causing a negative BOLD response. Because scopolamine does not attenuate the *negative BOLD response* in the prefrontal cortex, this effect is likely mediated by GABAergic inputs. (iv) Simultaneous incoming cholinergic inputs facilitate the GABAergic-mediated inhibitory effect on functional hyperemia, i.e., they basically just prolong the effect. Thus, an active cholinergic system modulates only the duration of the inhibitory effect on blood flow mediated by GABAergic inputs. If a BOLD response reflects changes in the concerted activity of local neuronal circuits, then the cholinergic system influences BOLD responses only when it modifies glutamatergic/GABAergic neuronal activity and not through a direct neuro-vascular coupling mechanism. It does not preclude neuronally released ACh from having hemodynamic effects, but these effects are small compared with glutamate- and GABA-mediated effects.

## Supplemental Material

sj-pdf-1-jcb-10.1177_0271678X211049820 - Supplemental material for The cholinergic system modulates negative BOLD responses in the prefrontal cortex once electrical perforant pathway stimulation triggers neuronal afterdischarges in the hippocampus Click here for additional data file. Supplemental material, sj-pdf-1-jcb-10.1177_0271678X211049820 for The cholinergic system modulates negative BOLD responses in the prefrontal cortex once electrical perforant pathway stimulation triggers neuronal afterdischarges in the hippocampus by Alberto Arboit, Karla Krautwald and Frank Angenstein in Journal of Cerebral Blood Flow & Metabolism
