# Supplementary material for: The cholinergic system modulates negative BOLD responses in the prefrontal cortex once electrical perforant pathway stimulation triggers neuronal afterdischarges in the hippocampus
Source: J Cereb Blood Flow Metab. 2021 Sep 30;42(2):364–80. doi: 10.1177/0271678X211049820 (PMC8795231; doi:10.1177/0271678X211049820)
Supplement: Supplementary material [file Supplemental_Figures_R2.docx]

## Supplemental Figures


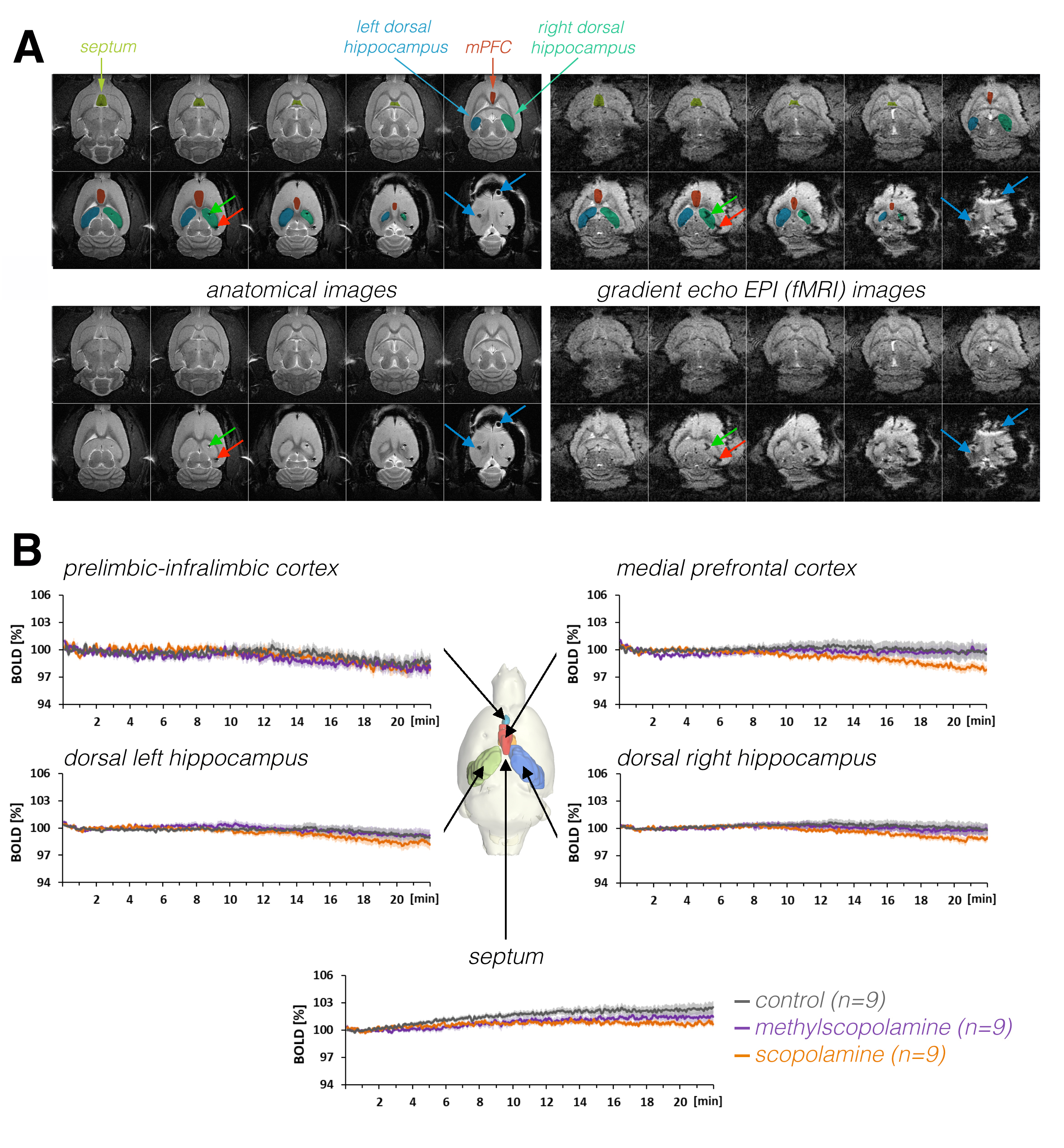


### Supplemental Figure S1

**A** Anatomical images (left) from rat brain. The arrows indicate the position of the recording electrode (green), stimulating electrodes (red), and grounding electrodes (blue). The artifacts generated by the electrodes are more pronounced in the corresponding gradient-echo EPI (right) that were used for fMRI. The location of the regions of interest (ROIs) used is shown in the top row. **B** BOLD time series in the right and left hippocampus, PrL-IL, mPFC, and septum were not modified by previous application of methylscopolamine (purple lines) or scopolamine (orange lines).


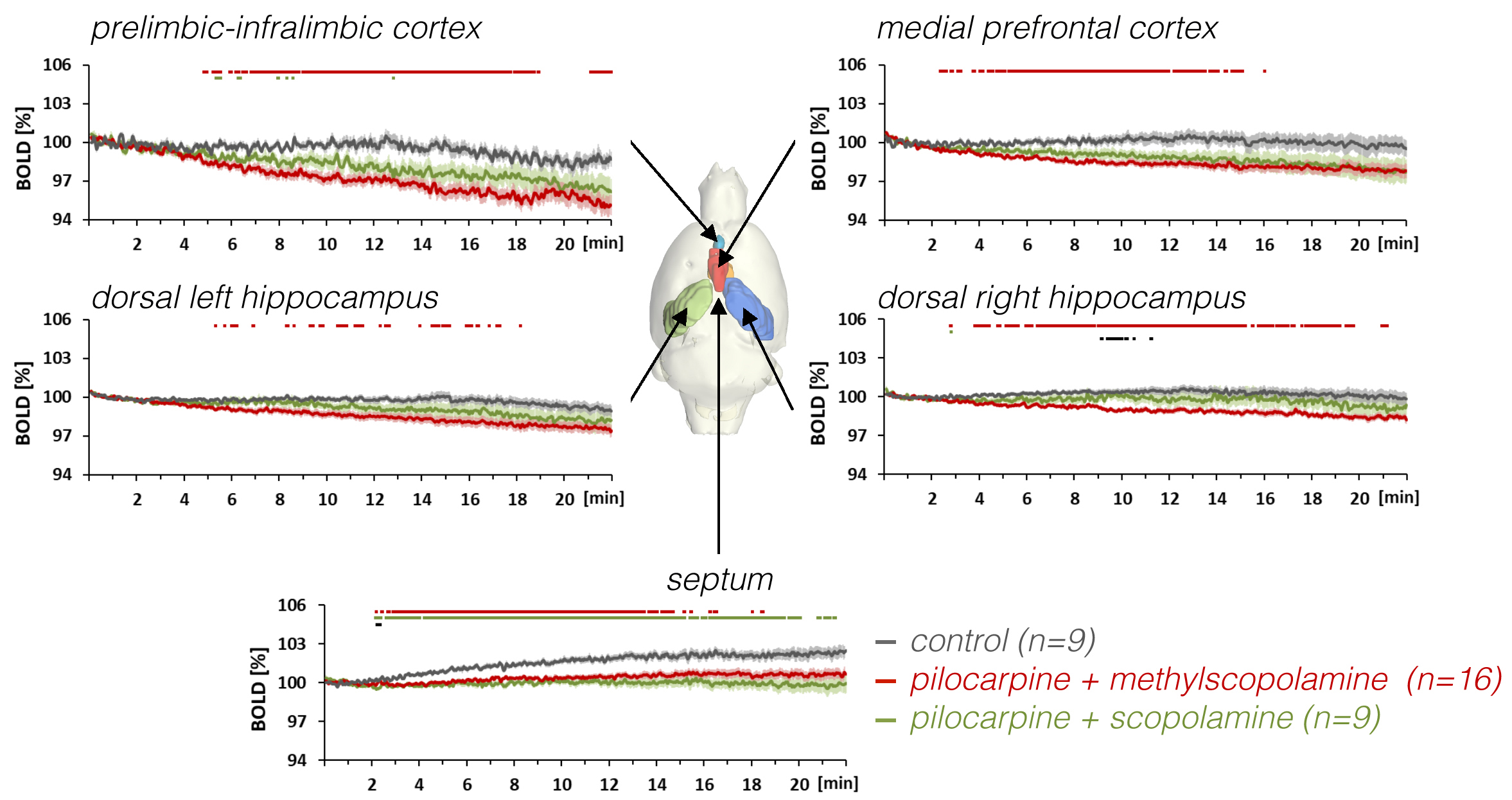


### Supplemental Figure S2

Development of baseline BOLD signals during an fMRI session without electrical stimulations. BOLD time series of five selected VOIs during a 22 min fMRI scan in the presence of pilocarpine and methylscopolamine (red lines), in the presence of pilocarpine and scopolamine (green lines), and the control condition (dark gray lines) are summarized. All lines represent the mean ± SD. Significant differences to the control condition are indicated as red or green bars.


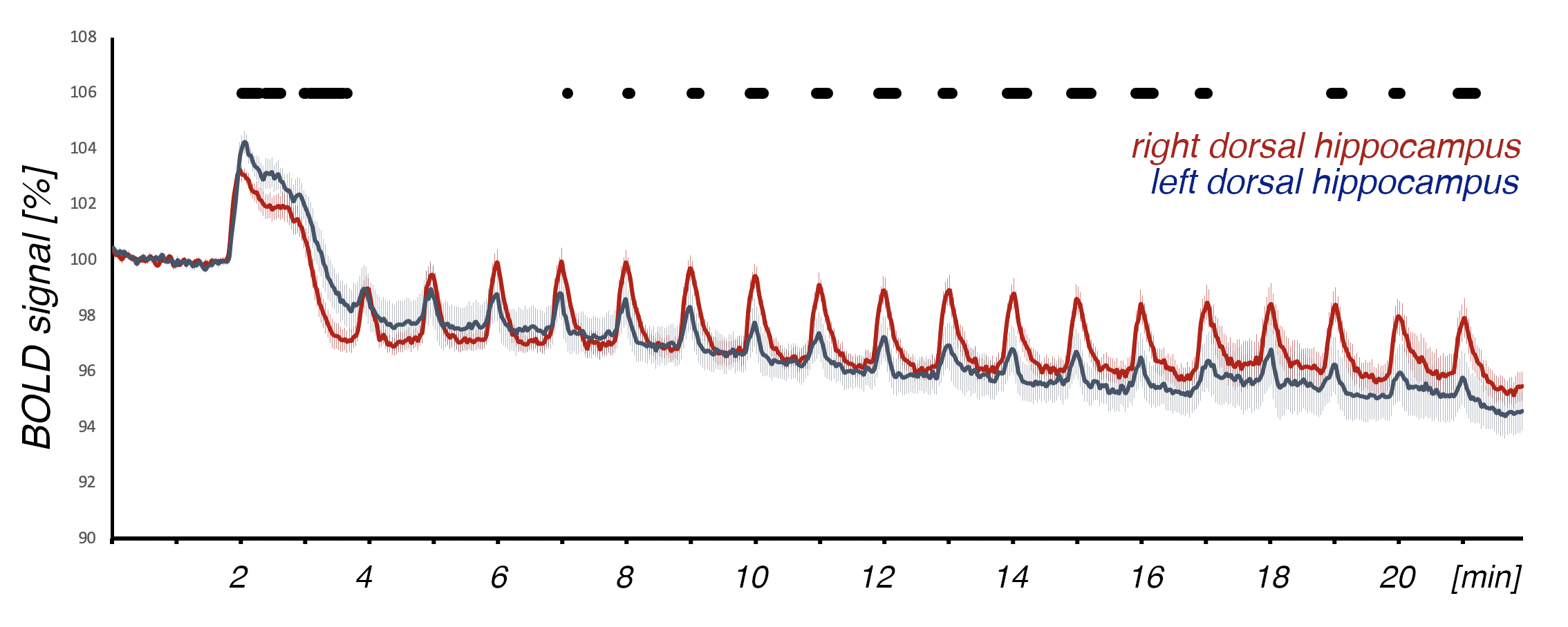


### Supplemental Figure S3

Development of BOLD signals in the right dorsal (red line) and left dorsal (blue line) hippocampus during repeated electrical stimulations of the right perforant pathway with high-frequency pulse bursts (n=17). The single stimulations resulted in significantly lower positive BOLD responses in the left dorsal hippocampus, but reduced baseline BOLD signals as much as in the right dorsal hippocampus. Significant differences in BOLD signals (p < 0.05) between the two BOLD time series are indicated by black dots.


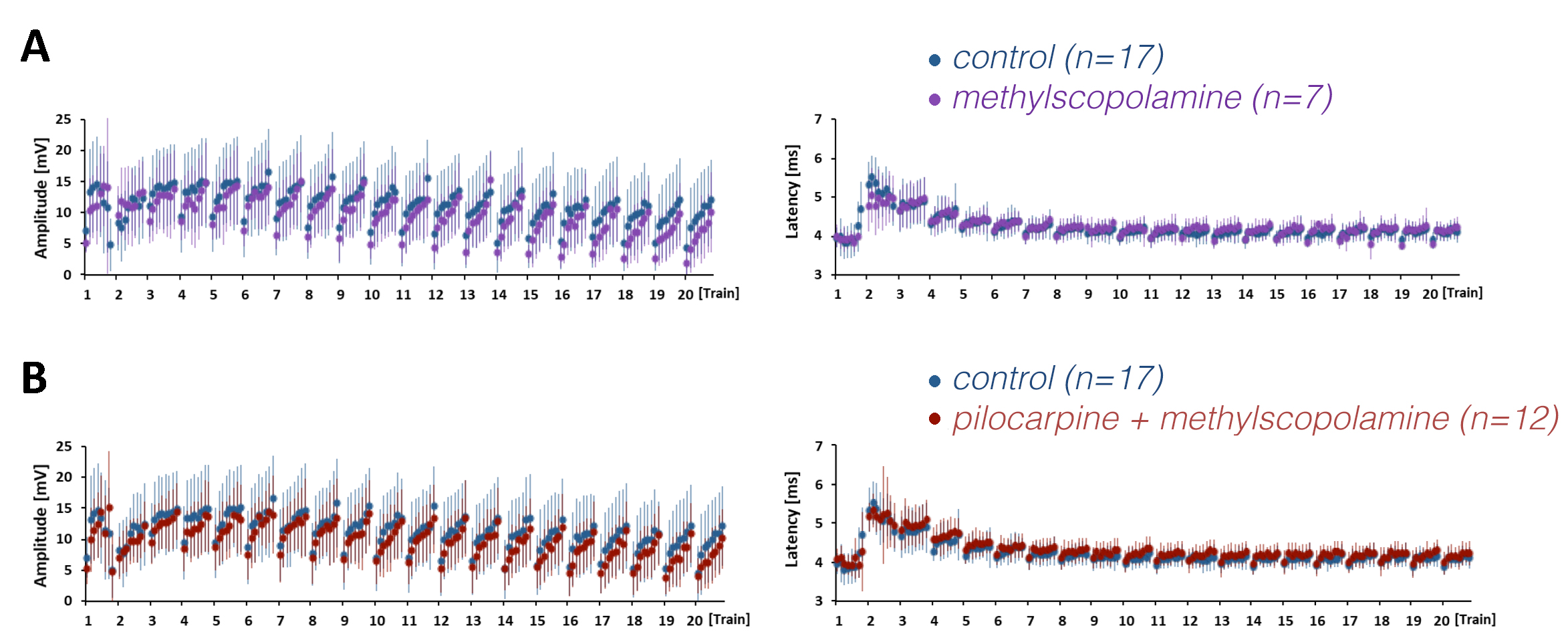


### Supplemental Figure S4

Summary of electrophysiological responses measured during fMRI experiments. **A** Methylscopolamine (purple dots) or **B** pilocarpine (in the presence of methylscopolamine, red dots) did not modify neuronal response in the dentate gyrus during 20 consecutive stimulation trains (control, blue dots).


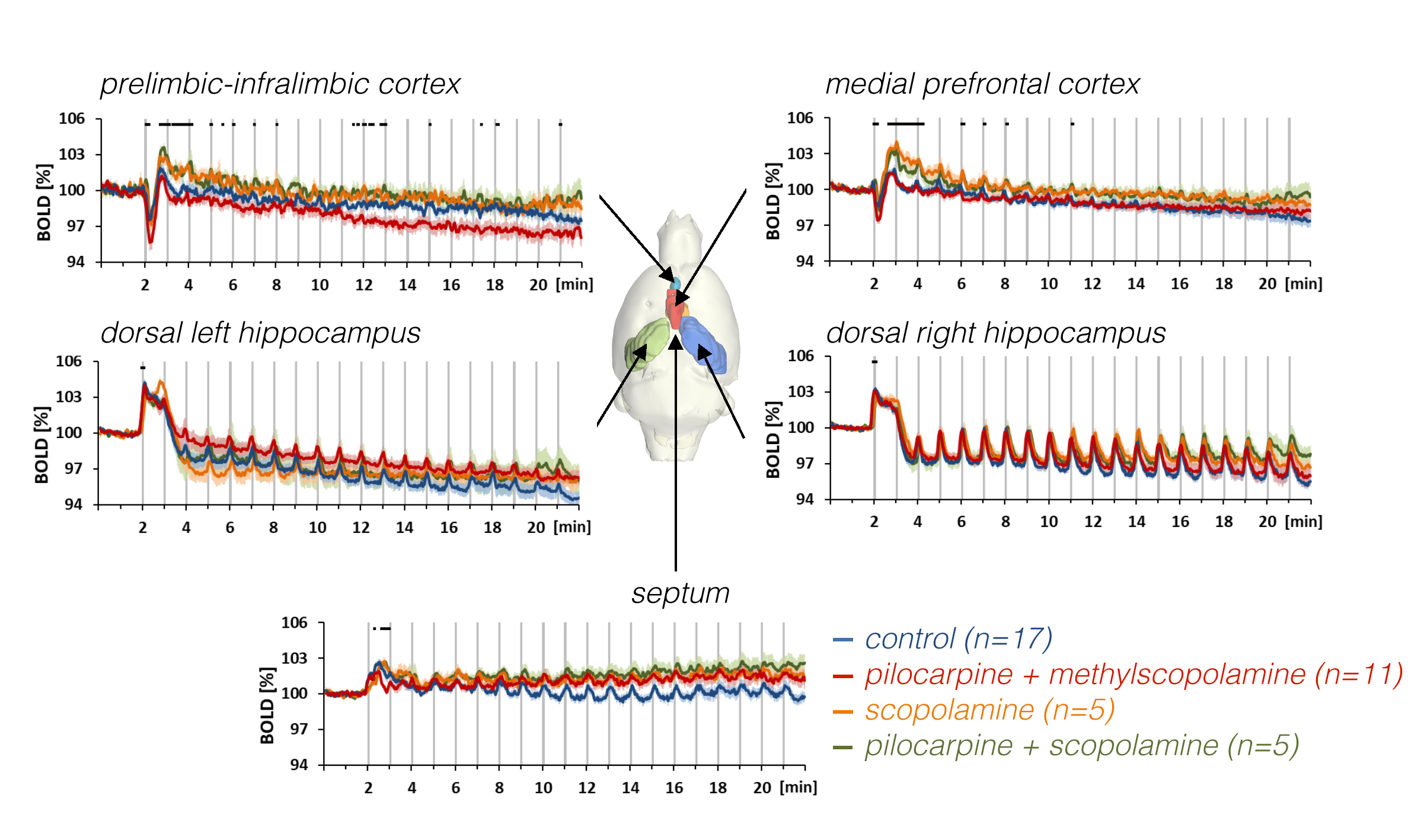


### Supplemental Figure S5

The effect of scopolamine on BOLD responses in the hippocampus, septum, and prefrontal cortex during stimulation of the right perforant pathway in the absence (yellow lines) or presence of pilocarpine (dark green lines). For comparisons, BOLD time series measured during stimulation of the perforant pathway in presence of pilocarpine alone (red lines) and during control conditions (blue lines) are added. Summarized are only stimulations that elicited h-nAD.


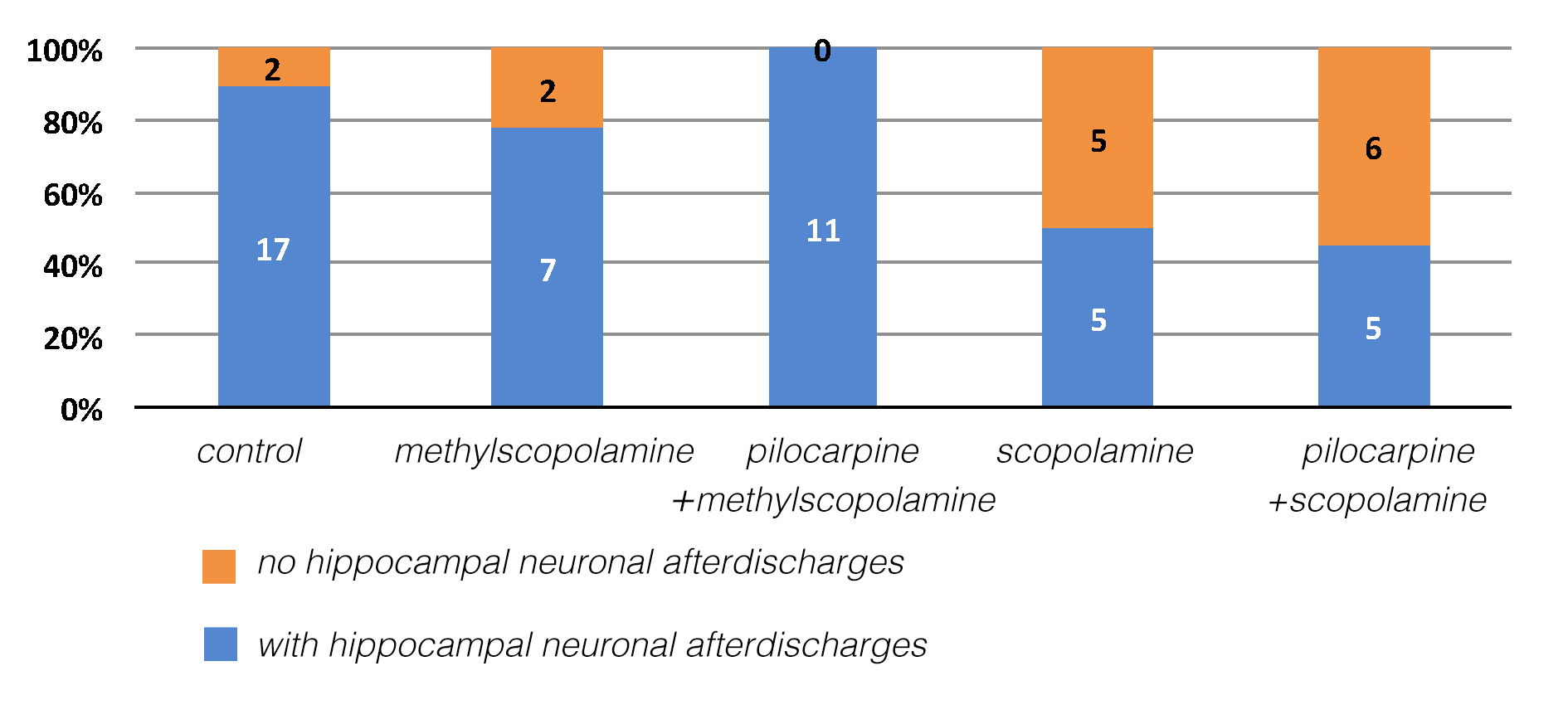


### Supplemental Figure S6

Incidence of h-nAD that were induced after the first stimulation period under different treatments. The number of animals that either elicited h-nAD (blue part of the columns) or did not elicited h-nAD (yellow part of the columns) are depicted for each condition. Note that the stimulation protocol was identical for all conditions.
