# Supplementary material for: The cholinergic system modulates negative BOLD responses in the prefrontal cortex once electrical perforant pathway stimulation triggers neuronal afterdischarges in the hippocampus
Source: J Cereb Blood Flow Metab. 2021 Sep 30;42(2):364–80. doi: 10.1177/0271678X211049820 (PMC8795231; doi:10.1177/0271678X211049820)
Supplement: Supplementary material [file Supplemental_Tables.docx]

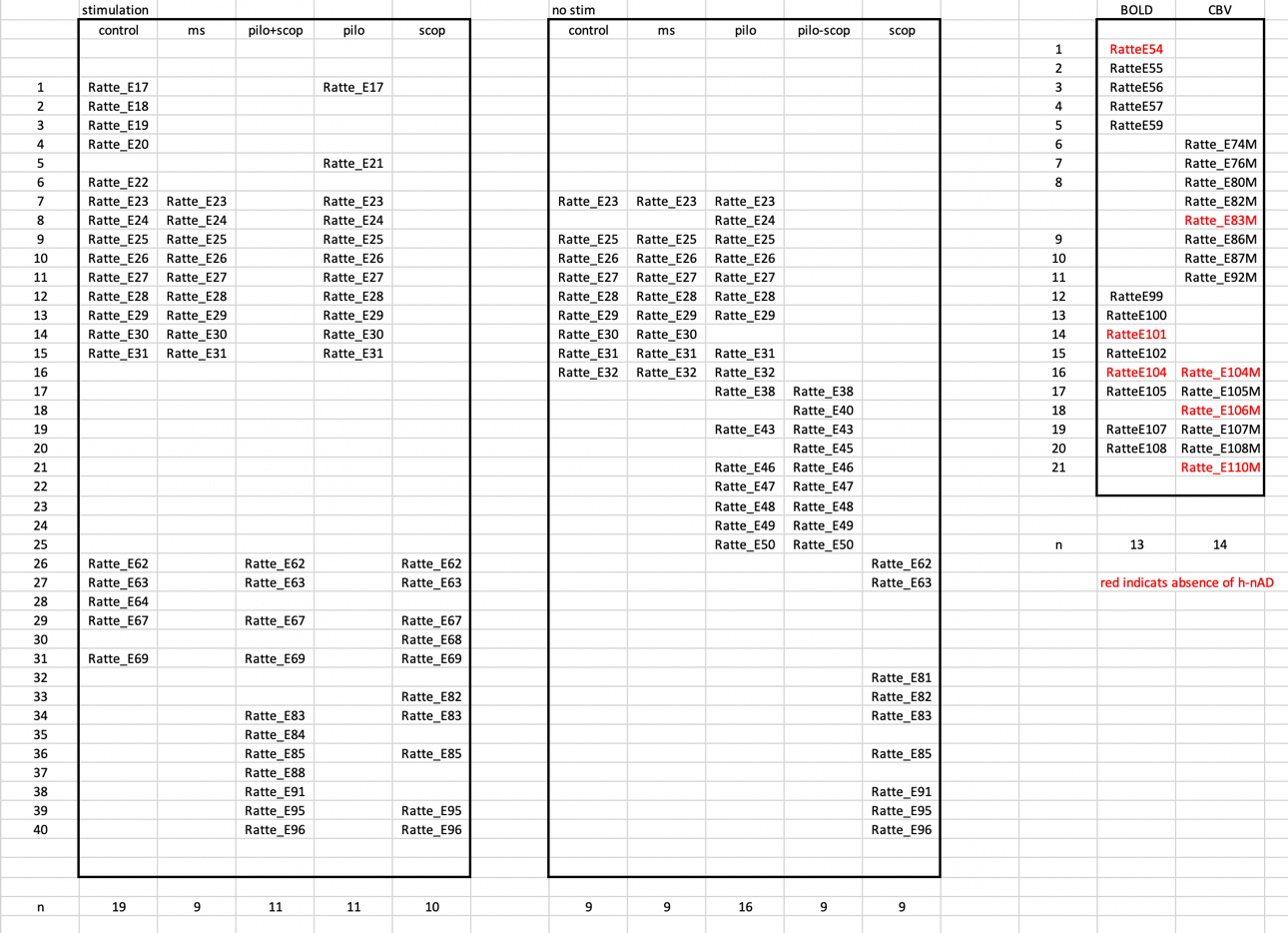


**Table S1.** Summary of all animals that were either repeatedly stimulated (left panel), or not stimulated (middle panel) in the presence or absence of a mACh receptor agonist/agonist. Right panel summarizes all animals that received only one stimulation train while either the BOLD or CBV response was measured (ms – methylscopolamine, pilo – pilocarpine, scop – scopolamine).

| regions |  | Correlation BOLD vs. CBV | t-test | p value  (2 sided) |
| --- | --- | --- | --- | --- |
| right dorsal  hippocampus | no nAD | 0.8146 | 15.7644 | 1.3 x 10^-31^ |
|  | with nAD | 0.3225 | 3.8239 | 0.0002 |
| medial prefrontal  cortex | no nAD | 0.4931 | 6.3635 | 3.3 x 10^-9^ |
|  | with nAD | 0.7933 | 14.6287 | 6.2 x 10^-29^ |
| septum | no nAD | 1.000 |  | 0 |
|  | with nAD | 0.4840 | 6.090 | 7.1 x 10^-9^ |

**Table S2.** Summary of correlations between BOLD and CBV signals after a stimulation period (period as shown in Figure 4). While in the right dorsal hippocampus the correlation between BOLD and CBV signals decreased as soon as h-nAD were induced, it increased in the prefrontal cortex. No significant changes in BOLD and CBV signals were detected in the septum in the absence of h-nAD, resulting in a correlation between these two parameters only when h-nAD was induced.
