# Supplementary material for: The cholinergic system modulates negative BOLD responses in the prefrontal cortex once electrical perforant pathway stimulation triggers neuronal afterdischarges in the hippocampus
Source: J Cereb Blood Flow Metab. 2021 Sep 30;42(2):364–80. doi: 10.1177/0271678X211049820 (PMC8795231; doi:10.1177/0271678X211049820)
Supplement: Supplementary file 1 [file sj-pdf-1-jcb-10.1177_0271678X211049820.pdf]

	stimulation					no stim				
	control	ms	pilo+scop	pilo	scop	control	ms	pilo	pilo-scop	scop
1	Ratte_E17			Ratte_E17						
2	Ratte_E18									
3	Ratte_E19									
4	Ratte_E20									
5				Ratte_E21						
6	Ratte_E22									
7	Ratte_E23	Ratte_E23		Ratte_E23		Ratte_E23	Ratte_E23	Ratte_E23		
8	Ratte_E24	Ratte_E24		Ratte_E24				Ratte_E24		
9	Ratte_E25	Ratte_E25		Ratte_E25		Ratte_E25	Ratte_E25	Ratte_E25		
10	Ratte_E26	Ratte_E26		Ratte_E26		Ratte_E26	Ratte_E26	Ratte_E26		
11	Ratte_E27	Ratte_E27		Ratte_E27		Ratte_E27	Ratte_E27	Ratte_E27		
12	Ratte_E28	Ratte_E28		Ratte_E28		Ratte_E28	Ratte_E28	Ratte_E28		
13	Ratte_E29	Ratte_E29		Ratte_E29		Ratte_E29	Ratte_E29	Ratte_E29		
14	Ratte_E30	Ratte_E30		Ratte_E30		Ratte_E30	Ratte_E30			
15	Ratte_E31	Ratte_E31		Ratte_E31		Ratte_E31	Ratte_E31	Ratte_E31		
16						Ratte_E32	Ratte_E32	Ratte_E32		
17								Ratte_E38	Ratte_E38	
18									Ratte_E40	
19								Ratte_E43	Ratte_E43	
20									Ratte_E45	
21								Ratte_E46	Ratte_E46	
22								Ratte_E47	Ratte_E47	
23								Ratte_E48	Ratte_E48	
24								Ratte_E49	Ratte_E49	
25								Ratte_E50	Ratte_E50	
26	Ratte_E62		Ratte_E62		Ratte_E62					Ratte_E62
27	Ratte_E63		Ratte_E63		Ratte_E63					Ratte_E63
28	Ratte_E64									
29	Ratte_E67		Ratte_E67		Ratte_E67					
30					Ratte_E68					
31	Ratte_E69		Ratte_E69		Ratte_E69					
32										Ratte_E81
33					Ratte_E82					Ratte_E82
34			Ratte_E83		Ratte_E83					Ratte_E83
35			Ratte_E84							
36			Ratte_E85		Ratte_E85					Ratte_E85
37			Ratte_E88							
38			Ratte_E91							Ratte_E91
39			Ratte_E95		Ratte_E95					Ratte_E95
40			Ratte_E96		Ratte_E96					Ratte_E96
n	19	9	11	11	10	9	9	16	9	9

	BOLD	CBV
1	RatteE54	
2	RatteE55	
3	RatteE56	
4	RatteE57	
5	RatteE59	
6		Ratte_E74M
7		Ratte_E76M
8		Ratte_E80M
		Ratte_E82M
		Ratte_E83M
9		Ratte_E86M
10		Ratte_E87M
11		Ratte_E92M
12	RatteE99	
13	RatteE100	
14	RatteE101	
15	RatteE102	
16	RatteE104	Ratte_E104M
17	RatteE105	Ratte_E105M
18		Ratte_E106M
19	RatteE107	Ratte_E107M
20	RatteE108	Ratte_E108M
21		Ratte_E110M
n	13	14

red indicats absence of h-nAD

<u>Table S1.</u> Summary of all animals that were either repeatedly stimulated (left panel), or not stimulated (middle panel) in the presence or absence of a mACh receptor agonist/agonist. Right panel summarizes all animals that received only one stimulation train while either the BOLD or CBV response was measured (ms – methylscopolamine, pilo – pilocarpine, scop – scopolamine).

regions		Correlation BOLD vs. CBV	t-test	p value (2 sided)
right dorsal hippocampus	no nAD	0.8146	15.7644	1.3×10^{-31}
	with nAD	0.3225	3.8239	0.0002
medial prefrontal cortex	no nAD	0.4931	6.3635	3.3×10^{-9}
	with nAD	0.7933	14.6287	6.2×10^{-29}
septum	no nAD	1.000		0
	with nAD	0.4840	6.090	7.1×10^{-9}

Table S2. Summary of correlations between BOLD and CBV signals after a stimulation period (period as shown in Figure 4). While in the right dorsal hippocampus the correlation between BOLD and CBV signals decreased as soon as h-nAD were induced, it increased in the prefrontal cortex. No significant changes in BOLD and CBV signals were detected in the septum in the absence of h-nAD, resulting in a correlation between these two parameters only when h-nAD was induced.
